# Co-Creation, Translation, and Localization of a Trauma-Informed Digital Mental Health Intervention for Frontline Workers: Protocol for a Multi-Country Feasibility Study

**DOI:** 10.2196/83521

**Published:** 2026-04-29

**Authors:** Nicola Cogan, Alison Kirk, Ian Couper, Johannes DeKock, William Hodgson, Susan Rasmussen, Spence Whittaker

**Affiliations:** 1 Department of Psychological Sciences and Health, University of Strathclyde, Graham Hills Building, 40 George Street, Glasgow, G1 1QE, United Kingdom; 2 Division of Rural Health (Ukwanda), Department of Global Health, University of Stellenbosch, Ukwanda, South Africa; 3 NHS Highlands, Inverness, United Kingdom

**Keywords:** digital mental health, co-creation, localization, translation, trauma-informed, frontline workers, participatory research, global mental health

## Abstract

**Background:**

Frontline professionals are routinely exposed to acute and cumulative occupational stressors that are associated with an elevated risk of psychological distress, burnout, and trauma-related difficulties. Digital mental health interventions offer scalable and flexible approaches to supporting psychological well-being in high-demand occupational environments. However, there remains limited empirical evidence regarding the feasibility and cultural adaptation of trauma-informed digital interventions across diverse international contexts.

**Objective:**

This protocol describes a multi-country feasibility study to co-create, translate, localize, and evaluate the usability and acceptability of Sentinel, a trauma-informed digital mental health intervention designed for frontline and trauma-exposed occupational groups.

**Methods:**

The study will be conducted across implementation sites in Saudi Arabia, South Africa, and Ukraine. Sentinel is delivered as a self-guided, modular digital intervention structured around 4 conceptual domains: recognize, remedy, recover, and resilience. Preparatory phases include stakeholder engagement, linguistic validation, and contextual localization. A 6-week single-arm feasibility pilot will be undertaken with approximately 100 frontline professionals recruited at each international site. Primary feasibility outcomes will focus on usability and acceptability, assessed through engagement analytics, Mobile App Rating Scale scores, and qualitative user feedback. Secondary exploratory outcomes will include preliminary indicators of psychological well-being, perceived psychological safety, and coping responses. Feasibility progression criteria will include recruitment and retention thresholds, patterns of intervention engagement, usability ratings, and qualitative indicators of cultural relevance to inform optimization prior to future controlled evaluation.

**Results:**

Preparatory study activities commenced in early 2026, including the development of international research partnerships and planning for stakeholder engagement and translation procedures. Ethical approval applications are scheduled for submission between June and September 2026. Participant recruitment is anticipated to begin in October 2026, with feasibility pilot data collection expected between November 2026 and March 2027. Quantitative and qualitative analyses are planned for April to June 2027. No outcome data are available at the time of manuscript submission.

**Conclusions:**

This study will generate evidence regarding the feasibility, usability, and cultural adaptation of a trauma-informed digital mental health intervention for frontline professionals across diverse socio-cultural settings. Findings will inform the iterative refinement of the intervention and guide progression to future effectiveness trials and wider implementation.

## Introduction

Co-creation processes that involve patients and the public have been shown to improve the design and implementation of health interventions by ensuring they are user centered and culturally appropriate [1]. Within digital health research, the involvement of end users can help identify barriers to adoption and tailor interventions to the needs of diverse communities [2]. Integrating patient and public involvement (PPI) in digital mental health research has also been associated with increased trust, enhanced relevance, and greater sustainability of interventions [3]. Frontline and trauma-exposed occupational groups globally experience elevated levels of psychological distress, burnout, and reduced well-being [4-6]. These individuals, including emergency responders, health and social care professionals, and essential service workers, often operate in high-pressure environments where exposure to trauma, moral injury, and cumulative stress is common [7]. Despite the substantial mental health burden, many frontline workers encounter barriers to accessing traditional face-to-face psychological support. These include stigma surrounding help-seeking, concerns about professional consequences, confidentiality issues, irregular working patterns, and limited availability of services, particularly in underresourced or rural settings [6,8,9]. In addition, existing support services are not always tailored to the cultural, occupational, and linguistic needs of frontline professionals, which can further reduce accessibility and engagement [10].

Digital mental health interventions offer the potential to provide timely, scalable, and flexible psychological support, particularly when designed to reflect the lived realities of frontline work [11]. However, for such interventions to be meaningful and usable across diverse socioeconomic and cultural contexts, they must be co-designed with end users and adapted to local cultural and linguistic environments [12]. Co-production with stakeholders is increasingly recognized as a key strategy for improving engagement and implementation in digital mental health [13], yet practical guidance on how to operationalize co-creation and localization across international contexts remains limited [14]. Cultural and contextual adaptation is particularly important in settings characterized by linguistic diversity, differing health system infrastructures, and varying patterns of trauma exposure, all of which may influence how digital tools are perceived and used [3,15].

This protocol outlines a multi-site study designed to address these challenges through the co-creation, translation, and localization of Sentinel, a trauma-informed digital mental health intervention developed in collaboration with academic and clinical partners in the United Kingdom. The study aims to evaluate the usability, acceptability, and perceived psychological benefit of localized versions of the intervention among frontline professionals in diverse global contexts [16]. Initial testing of the intervention in the United Kingdom has now been completed with a sample of 100 frontline workers, and findings from this phase are currently being prepared for peer-reviewed publication. Saudi Arabia, South Africa, and Ukraine were selected as implementation sites to enable examination of digital mental health localization across settings characterized by differing health system priorities, linguistic landscapes, socio-political conditions, and patterns of occupational trauma exposure. Saudi Arabia represents a rapidly evolving health system context in which national policy initiatives, including Vision 2030, emphasize digital health innovation and workforce well-being [17]. South Africa provides a context marked by substantial linguistic diversity, socioeconomic inequality, and high levels of trauma exposure among public-facing occupational groups [18]. Ukraine offers a conflict-affected setting in which frontline professionals may experience cumulative and acute stress within disrupted service infrastructures and humanitarian conditions [19]. These sites provide an opportunity to explore how culturally responsive digital mental health interventions can be adapted across heterogeneous global environments. This comparative approach supports examination of localization processes in relation to cultural norms, occupational demands, and health system readiness, thereby contributing to theoretical and practical understanding of scalable trauma-informed digital support tools.

This study aims to develop and evaluate a culturally adapted digital mental health intervention for frontline and trauma-exposed occupational groups across diverse international contexts. The objectives are as follows:

1. To co-create culturally relevant content for the Sentinel digital mental health intervention by engaging local stakeholders in Saudi Arabia, South Africa, and Ukraine. This collaborative approach ensures the intervention aligns with the unique cultural, linguistic, and occupational contexts of these regions.
2. To translate and linguistically validate the Sentinel intervention into Arabic, isiXhosa, Afrikaans, and Ukrainian. This process will adhere to established best practices, including forward-backward translation, expert panel reviews, and pretesting with target populations to ensure semantic, conceptual, and experiential equivalence.
3. To conduct a 6-week feasibility study to primarily assess the usability and acceptability of the localized versions of the Sentinel intervention. Usability and acceptability will be operationalized through user engagement metrics, Mobile App Rating Scale (MARS) scores, and qualitative user feedback. Secondary exploratory outcomes will include preliminary indicators of psychological well-being, perceived psychological safety, and behavioral coping responses.
4. To evaluate the impact of localization on user engagement and cultural fit by analyzing user interaction data, conducting qualitative interviews, and assessing cultural relevance through participant feedback. Insights gained will inform iterative refinements of the intervention and contribute to broader understandings of strategies for culturally adapting digital mental health tools.

## Methods

### Study Design

This protocol places a strong emphasis on PPI as a foundational element of the co-creation design process [1]. Engaging with frontline professionals and local stakeholders ensures that cultural nuances, lived experiences, and context-specific needs are embedded from the outset. PPI will be integrated throughout the project lifecycle, from the design of localized content to feedback on app usability and dissemination of findings. Meaningful PPI enhances the relevance, accessibility, and uptake of digital mental health interventions, particularly among underserved and trauma-exposed populations (Table 1).

**Table 1.** Summary of patient and public involvement (PPI) across study phases.

Study phase	PPI activities	Purpose	Participant groups involved
Phase 1: co-design and cultural insight	Participation in cultural insight workshops and needs assessments	Identify culturally relevant mental health concepts, language, and delivery preferences	Frontline workers, community leaders, and mental health advocates
Phase 2: translation and validation	Review of translated materials and contribution to terminology selection	Ensure linguistic equivalence, cultural resonance, and avoidance of stigmatizing or clinical jargon	Bilingual PPI contributors and local health professionals
Phase 3: localization and user testing	Testing of prototype versions of the app and usability feedback through interviews	Refine visual and functional elements and assess cultural and contextual fit	Frontline professionals and peer supporters
Phase 4: evaluation and refinement	Participation in posttrial qualitative interviews and focus groups	Explore lived experiences of using the app and identify areas for improvement	Study participants and local advisory panel members
Dissemination	Co-creation of community-friendly summaries and input on academic and policy outputs	Promote accessibility of findings and ensure relevance to target audiences	PPI contributors, local organizations, and NGO^a^ partners

a NGO: nongovernmental organization.

This study uses a multi-phase, mixed-methods, participatory research design [20] to develop, localize, and evaluate the Sentinel digital mental health intervention across diverse cultural contexts. This approach supports the iterative adaptation of digital interventions to meet the nuanced needs of frontline and trauma-exposed populations, ensuring that both content and delivery are grounded in real-world experiences and local contexts.

### The Sentinel Intervention

Sentinel is a modular, trauma-informed digital mental health intervention designed to provide accessible psychological support for frontline and trauma-exposed professionals. The intervention is delivered as a self-guided mobile-optimized platform, enabling users to engage independently with content without structured therapist or facilitator involvement. While the app may be introduced within organizational well-being initiatives, ongoing engagement is intended to be flexible and responsive to individual psychological needs and occupational demands. The intervention is informed by an integrative theoretical framework incorporating principles derived from cognitive behavioral approaches, mindfulness-based stress regulation, and strengths-focused psychological models. This blended orientation aims to support both immediate emotional and physiological regulation and longer-term reflective processes associated with adaptive coping and psychological resilience in high-stress work environments.

Sentinel includes a range of on-demand interactive components delivered through multi-modal formats, including brief text-based content, audio-guided exercises, and visual elements. Core features include short guided practices such as breathing regulation strategies, grounding exercises, and mindfulness-based attentional refocusing techniques designed for independent use during periods of acute stress. Users may also access psychoeducational modules addressing topics relevant to frontline occupational well-being, including trauma exposure, burnout, moral injury, sleep disruption, and psychological safety. Reflective activities are incorporated to support awareness of coping responses, emotional processing, and strengths development over time.

Intervention content is organized within a conceptual “4 Rs” framework comprising recognize, remedy, recover, and resilience. These domains represent key psychological processes relevant to managing acute and cumulative occupational stress. The *recognize* domain focuses on enhancing awareness of internal stress responses and contextual indicators of psychological strain. The *remedy* domain provides brief, in-the-moment strategies aimed at regulating physiological arousal and supporting immediate coping. The *recover* domain emphasizes restorative processes such as sleep hygiene, self-care practices, and structured reflection on challenging experiences. The *resilience* domain supports longer-term adaptive coping, strengths use, and sustained psychological functioning.

The intervention includes a digital library of over 70 individual components that users can access flexibly and nonsequentially according to personal need and working patterns. A personalized dashboard enables users to track engagement, revisit previously accessed resources, and reflect on evolving coping strategies. The intervention is positioned as a preventative and supportive resource and is not intended to function as a diagnostic or crisis response tool. During localization and feasibility testing, selected content elements and interface features may be iteratively refined to enhance contextual relevance, cultural acceptability, and usability across implementation settings while maintaining fidelity to core trauma-informed principles.

### Project Phases

#### Overview

The Sentinel research and implementation program is structured across 4 sequential but overlapping phases, designed to support iterative development, contextual adaptation, and evaluation of the digital intervention. This phased approach reflects established guidance for the development and testing of complex interventions and digital mental health technologies.

#### Phase 1: Stakeholder Engagement and Cultural Insight Workshops

In this initial phase, stakeholder engagement workshops will be held with frontline professionals and local advisors in Saudi Arabia, South Africa, and Ukraine. These sessions will gather in-depth qualitative insights into local mental health needs, existing coping practices, help-seeking barriers, and cultural and language norms. This information will guide decisions regarding content framing, language tone, visual design, and cultural sensitivities to ensure contextual relevance.

#### Phase 2: Translation and Linguistic Validation

Building on the insights from phase 1, the intervention will be professionally translated into Arabic, isiXhosa, Afrikaans, and Ukrainian. The process will follow international best practices for health translation, including forward-backward translation, reconciliation by expert panels, and cognitive pretesting with representatives from the target user groups. This phase ensures not only semantic and grammatical accuracy but also conceptual alignment with local understandings of mental health and trauma.

#### Phase 3: Localization and User Adaptation

This phase focuses on adapting the Sentinel interface and content based on linguistic validation outcomes and additional cultural input. Localized modules will be tested and iteratively refined in collaboration with end users. Adaptations may include changing idioms, modifying imagery, adjusting visual layout, or incorporating locally relevant metaphors. The localization process will be guided by user-centered design principles, ensuring that the platform is intuitive, engaging, and aligned with the day-to-day realities of its intended users.

#### Phase 4: Pilot Feasibility Trial and Posttrial Qualitative Interviews

A 6-week pilot feasibility study will be conducted in each implementation site. The primary aim of this phase is to evaluate the usability and acceptability of the localized Sentinel intervention rather than to determine clinical effectiveness. Participants will receive access to the localized version of the app and will be invited to engage with its features in a self-directed manner. Primary feasibility outcomes will include user engagement metrics (eg, frequency and duration of use and interaction with core features), MARS scores, and qualitative feedback on user experience and perceived cultural relevance. Secondary exploratory outcomes will include preliminary indicators of psychological well-being, perceived psychological safety, and behavioral coping responses, assessed using validated pre- and post-intervention measures. In-app analytics will provide additional data on engagement patterns, while semi-structured posttrial interviews will explore acceptability, cultural fit, and suggestions for refinement. Findings from this phase will inform progression decisions and optimization of the intervention prior to any future effectiveness evaluation. As this feasibility study does not include a control or comparison group, psychological outcomes will be interpreted cautiously as feasibility signals rather than evidence of intervention effectiveness.

### Setting

The study will be conducted across 3 international sites: Saudi Arabia, South Africa, and Ukraine, each involving regional research teams and community health partners. This collaborative, community-engaged approach ensures that the Sentinel digital mental health intervention is culturally adapted and contextually relevant for frontline and trauma-exposed occupational groups in each location.

In each country, regional research teams will work closely with community health partners, including local clinicians, mental health practitioners, and representatives from frontline services. These partnerships facilitate the co-creation of intervention content, ensuring that it aligns with the specific cultural, linguistic, and occupational contexts of the target populations. Engaging local stakeholders in this manner enhances the intervention’s acceptability, as it incorporates the lived experiences and insights of those directly involved in frontline work [21]. This approach aligns with principles of community-based participatory research, which emphasize equitable collaboration between researchers and community members throughout the research process [22]. By involving community health partners in all phases of the study, from design and implementation to evaluation and dissemination, the research fosters mutual learning and capacity building, ultimately contributing to more sustainable and impactful health interventions [23]. The international scope of the study allows for comparative analyses across diverse settings, providing insights into how cultural and contextual factors influence the implementation and outcomes of digital mental health interventions. This knowledge will inform the development of best practices for adapting such interventions to various global contexts, thereby enhancing their scalability in addressing the mental health needs of frontline workers worldwide [24].

### Participants

#### Overview

The study aims to recruit 100 participants at each of the 3 international sites, that is, Saudi Arabia, South Africa, and Ukraine, resulting in a total sample size of 300 participants. The proposed sample size is consistent with recommendations for feasibility and pilot studies and aligns with the UK Medical Research Council framework for the development and evaluation of complex interventions, which emphasizes the importance of adequately powered feasibility work to inform progression to later-stage effectiveness trials. The primary purpose of this sample is therefore to enable estimation of recruitment and retention rates, assessment of usability and acceptability outcomes, and preliminary parameter estimation to support future trial design and power calculations. The target of approximately 100 participants per site is also informed by recruitment achieved in a recent UK-based feasibility study of the Sentinel intervention, which has successfully enrolled a comparable sample and is scheduled for completion in August 2026. As such, the planned sample is considered appropriate for evaluating implementation processes, engagement variability, and contextual feasibility across diverse international settings rather than for detecting statistically significant clinical effects.

#### Inclusion Criteria

For the purposes of this study, frontline professionals are operationally defined as individuals working in occupations characterized by direct and sustained exposure to potentially traumatic events, high emotional demand, or cumulative occupational stress. This includes, but is not limited to, health care workers, emergency responders, humanitarian personnel, social care practitioners, and essential service workers in high-intensity public-facing roles. Participants must be aged 18 years or older, proficient in the local language(s) of the respective study site, and actively used or engaged in frontline professional practice during the study period, with a minimum of 6 months of experience in their current role. Eligibility will additionally require self-reported exposure within the preceding 12 months to acute traumatic incidents (eg, exposure to death, serious injury, and crisis situations), repeated high-stress operational demands, or cumulative work-related psychological strain associated with frontline duties. Contextual information regarding occupational role, service setting, length of service, and typical patterns of trauma or stress exposure will be collected to support descriptive reporting and exploratory sub-group analyses across implementation sites. To support inclusivity and equitable participation, efforts will be made to secure funding for digital devices or connectivity resources for individuals who may otherwise face barriers to accessing the intervention or study procedures.

#### Exclusion Criteria

Individuals will be excluded from the study if they are experiencing an acute mental health crisis that requires immediate clinical intervention. This criterion is established to prioritize participant safety, as the Sentinel intervention is not designed to address acute psychiatric emergencies.

### Recruitment

Recruitment will be conducted through a combination of online and offline methods to ensure broad reach and inclusivity [25]. Online strategies may include targeted advertisements on social media platforms, professional networks, and organizational websites. Offline recruitment may involve collaboration with local organizations, distribution of informational materials, and direct outreach to potential participants through professional associations and community events. This multi-faceted approach aims to engage a diverse cohort of frontline professionals, reflecting the varied populations that the Sentinel intervention is intended to serve.

By using these inclusion and exclusion criteria and comprehensive recruitment strategies, the study seeks to assemble a representative sample of frontline professionals to evaluate the cultural adaptability of the Sentinel digital mental health intervention.

### Measures

The study will, via Qualtrics (Qualtrics LLC), present several validated measures (Table 2), including the Short Warwick Edinburgh Mental Well-being Scale; Depression, Anxiety, Stress Scale-21; Burnout Measure-Short Version; Post-Traumatic Growth Inventory; MARS; Neuroception of Psychological Safety Scale; International Physical Activity Questionnaire–Short Form; and qualitative interviews after intervention. All psychometric tools and interview guides will be translated and culturally adapted for use in each implementation site, following best practice for forward-backward translation and validation to ensure linguistic accuracy and conceptual equivalence. This will support meaningful cross-cultural comparisons and ensure the relevance and acceptability of the tools across diverse occupational and socio-cultural contexts.

Progression criteria will include pre-defined thresholds for recruitment (>60% target), retention (>50% completion), mean MARS usability ratings (>3.5), and qualitative evidence of cultural acceptability. These criteria will inform optimization decisions prior to any definitive effectiveness evaluation.

**Table 2.** Study measures.

Measure	Description	Questions, n	Authors
Short Warwick Edinburgh Mental Well-being Scale	Measures subjective mental well-being	7	[26]
Depression, Anxiety, Stress Scale-21	Evaluates mental health distress	21	[27]
Burnout Measure-Short Version	Assesses burnout across diverse occupational and community contexts	21	[28]
Post-Traumatic Growth Inventory	Measures posttraumatic growth	10	[29]
Mobile App Rating Scale	Assesses the quality of mobile health apps	29	[30]
Neuroception of Psychological Safety Scale	Captures psychological safety	29	[31]
International Physical Activity Questionnaire–Short Form	Assesses intensity of physical activity and sitting time	7	[32]

### Translation and Localization Process

The localization of the Sentinel digital mental health intervention will incorporate culturally and linguistically tailored adaptations for Arabic-speaking, South African (isiXhosa and Afrikaans), and Ukrainian frontline professionals.

For the Arabic version, the translation process is ongoing and will incorporate co-designed terminology for trauma-related concepts, ensuring that psychological terms resonate with the target audience’s cultural and linguistic context. This approach acknowledges that direct translations may not capture the nuances of trauma experiences in Arabic-speaking cultures [33]. Additionally, local idioms for stress, safety, and body-based practices will be integrated to make the intervention more relatable and meaningful [34]. The interface and content will be adapted to accommodate right-to-left formatting, aligning with the reading habits of Arabic-speaking users [35]. Visual elements will be selected and designed to reflect cultural norms, values, and environments familiar to the target audience (eg, appropriate representation of regional dress, settings, and tone), enhancing relatability and engagement.

In South Africa, the adaptation will include translations into English, Afrikaans, and isiXhosa, recognizing the linguistic diversity of the region. The content will incorporate local narratives and collectivist metaphors, reflecting the communal experiences and values prevalent in South African societies [36]. Similar to the adaptations for Arabic-speaking users, commensurate visual adaptations will be made to represent regional variations for South African users, ensuring that the intervention resonates with the users’ daily lives and professional contexts.

The Ukrainian adaptation will involve the use of trauma-sensitive language that reflects current socio-political realities, acknowledging the unique challenges faced by Ukrainian frontline professionals. Careful translation of psychosocial concepts will be undertaken to ensure cultural and clinical accuracy. Visual design will be consistent with regional aesthetics and norms, enhancing the intervention’s relevance. Translation efforts will involve collaboration between mental health professionals and linguists to ensure that the content is both culturally appropriate and clinically sound. By addressing these linguistic and cultural considerations, the Sentinel intervention aims to provide accessible, relevant, and meaningful support for the mental health of frontline professionals across diverse populations. This culturally sensitive approach is essential for ensuring that digital mental health interventions are both usable and impactful in varied contexts.

### Ethical Considerations

Ethical approval for the study will be obtained from the relevant institutional and/or provincial research ethics committees in each participating country (Saudi Arabia, South Africa, and Ukraine) prior to the commencement of any study procedures. Ethical governance will be implemented in accordance with country-specific regulatory requirements at each implementation site. In Saudi Arabia, approval will be sought through appropriate institutional or regional ethics review bodies in collaboration with local academic and clinical partners supporting co-creation and pilot implementation. In Ukraine, governance processes will be facilitated through partnerships with academic and humanitarian health organizations to ensure compliance with national ethical review procedures and data protection regulations. These locally embedded collaborations will support culturally appropriate oversight, participant safeguarding, and adherence to national research governance frameworks.

All procedures will align with international ethical standards, including the Declaration of Helsinki, and relevant local regulations [37]. Electronic informed consent will be obtained from all participants following provision of detailed information regarding study aims, procedures, potential risks and benefits, data management processes, and participants’ rights, including the right to withdraw without penalty. Quantitative survey data collected via Qualtrics will initially be pseudonymized using encrypted participant codes to allow withdrawal during the data collection phase. Following a 2-week post-collection period, datasets will be fully anonymized through the removal of identifiable information, after which withdrawal will no longer be possible. No financial or nonfinancial compensation will be provided to participants for participation in this study.

For qualitative interviews, participants will similarly be offered a 2-week withdrawal period following data collection. During this time, audio recordings will be securely stored and labeled using pseudonyms. Transcription and anonymization procedures, including removal of identifying details such as names or locations, will be undertaken only after the withdrawal window has elapsed. This approach balances participants’ autonomy and control over their data with the methodological integrity of qualitative analysis. Interviews may be conducted either in person or remotely depending on participant preference and local logistical considerations. A trauma-informed distress protocol will be implemented across both modalities to safeguard participant well-being. This will include procedures for recognizing signs of distress, pausing, or discontinuing interviews where necessary, providing immediate access to appropriate support resources, and conducting follow-up where indicated. Researchers involved in data collection will receive training in trauma-informed interviewing and cultural competence, alongside access to supervision and structured debriefing to mitigate potential vicarious distress.

All study data will be stored on encrypted, password-protected institutional servers, with access restricted to authorized research team members. Anonymization procedures will involve the systematic removal or coding of personal identifiers to prevent participant identification. These measures are consistent with recognized best practice in research data protection and confidentiality and ensure compliance with relevant ethical and legal standards [38]. The study design is underpinned by trauma-informed research principles to promote psychological safety for both participants and researchers [39]. Participation will be voluntary and designed to be empowering, with culturally relevant support resources available where required. Close collaboration with the PPI group will support contextual adaptation of distress protocols and ethical procedures across study settings. Embedding these ethical safeguards aims to uphold the highest standards of research integrity while generating meaningful evidence to inform the development and implementation of digital mental health interventions.

### Analysis and Data Monitoring

Quantitative data collected through standardized instruments (ie, Short Warwick Edinburgh Mental Well-being Scale; Depression, Anxiety, Stress Scale-21; Burnout Measure-Short Version; Post-Traumatic Growth Inventory; MARS; Neuroception of Psychological Safety Scale; and International Physical Activity Questionnaire–Short Form) will be analyzed using descriptive and inferential statistics to explore usability, acceptability, and preliminary psychological outcomes within and across study sites. Where appropriate, sub-group analyses may be conducted to identify contextual or demographic variations.

Qualitative data from post-intervention interviews will be analyzed using reflexive thematic analysis [40]. This approach is well-suited for exploring complex, context-dependent experiences and is grounded in an interpretivist paradigm. Where appropriate, secure artificial intelligence–assisted transcription tools may be used to support the initial processing of qualitative interview data. All transcripts will be reviewed, verified, and corrected by members of the research team to ensure accuracy and contextual fidelity. Translation and linguistic adaptation procedures will involve collaboration with bilingual researchers and culturally informed practitioners to ensure semantic and conceptual equivalence across study sites. All data processing procedures will comply with institutional data protection and confidentiality requirements. Transcripts will be read repeatedly for familiarization, then coded inductively using NVivo or equivalent software. Codes will be developed into themes through iterative reflection, with attention paid to both semantic and latent meanings. To ensure rigor, the research team will engage in reflexive journaling and regular analytic discussions to challenge assumptions and support theme development [41]. Cross-site comparisons will be used to explore shared and culturally specific experiences related to intervention use, engagement, and perceived impact. Given the nature of the study as a feasibility protocol for a digital mental health intervention, no formal Data Safety Monitoring Board will be established. However, internal monitoring procedures will be implemented [42]. Each country site will designate a local research coordinator responsible for overseeing adherence to the protocol, participant safety, and data integrity [43]. The lead study team at the University of Strathclyde will conduct monthly virtual check-ins with site teams to review progress, address challenges, and ensure consistency in data collection and reporting. All research activities will be subject to institutional ethical oversight, and any protocol deviations or adverse events will be documented and reported to the relevant ethics committees in accordance with local regulatory requirements.

Progression criteria for feasibility will be pre-defined to inform decisions regarding refinement of the intervention and progression to a future effectiveness trial. These criteria will include recruitment rates across sites, participant retention throughout the 6-week pilot period, levels of engagement with core app features, and thresholds indicating acceptable usability and satisfaction based on MARS scores and qualitative user feedback. Patterns of missing data, technological barriers, and contextual implementation challenges will also be reviewed as part of the feasibility assessment.

## Results

At the time of manuscript submission, preparatory study activities have commenced, including the establishment of international research partnerships, initial stakeholder engagement planning, and scoping of linguistic translation and cultural adaptation procedures across participating sites. Formal ethical approval applications are scheduled for submission between June and September 2026, with relevant institutional and regional review bodies in Saudi Arabia, South Africa, and Ukraine. Participant recruitment and implementation of the pilot feasibility study are planned to commence in October 2026, following completion of localization and linguistic validation procedures. Data collection for the 6-week feasibility pilot is expected to take place between November 2026 and March 2027, with quantitative and qualitative analyses scheduled for April to June 2027. No outcome data are available at this stage (Table 3).

**Table 3.** Timeline of study phases.

Phase	Activities	Timeline (mo)
1	Stakeholder engagement and cultural insight workshops	1‐3
2	Translation and linguistic validation	4‐6
3	Localization and user adaptation	7‐9
4	Pilot feasibility trial and posttrial interviews	10‐15

This phased timeline provides a structured framework for monitoring study implementation, feasibility progression, and coordination across participating international sites.

## Discussion

### Anticipated Findings

This protocol presents a comprehensive and participatory framework for the co-creation, translation, and localization of the Sentinel digital mental health intervention across three culturally and linguistically diverse settings. The methodology foregrounds community engagement and iterative design, acknowledging the importance of culturally relevant adaptations in enhancing the accessibility and usability of digital mental health tools [2,3]. Cultural adaptation of digital health interventions is increasingly recognized as essential for improving mental health outcomes among underserved populations, particularly in low- and middle-income countries and non-Western contexts [12,18]. Despite the growing emphasis on co-design and PPI, empirical guidance on operationalizing these processes in international digital health projects remains limited [13,14]. This study directly addresses that gap by outlining a structured and context-responsive approach to localization, grounded in the lived experiences of frontline professionals.

By prioritizing stakeholder engagement from the outset, the protocol operationalizes key principles of community-based participatory research [22], ensuring that local knowledge and socio-cultural insights are embedded within the intervention design. This approach not only enhances cultural resonance and acceptability but also promotes equity in health research and fosters greater ownership among users [21]. Furthermore, by incorporating linguistic validation procedures, such as forward-backward translation and expert panel reviews, the study aligns with international best practices for the translation of health interventions [44]. Importantly, the inclusion of trauma-informed principles throughout the study acknowledges the ethical imperative of minimizing harm in research with populations who may be experiencing ongoing stress or adversity [32]. The provision of researcher training and participant safeguarding protocols underscores a commitment to psychological safety, a particularly salient consideration in humanitarian and high-risk occupational contexts [6,19]. In addition to the primary feasibility outcomes, the study will provide valuable insights into how digital interventions are perceived and used in distinct cultural settings. Prior research suggests that visual and narrative elements that reflect local realities can significantly enhance user engagement and emotional connection with digital mental health tools [34,45]. By integrating culturally specific metaphors, idioms, and design elements, Sentinel seeks to move beyond simple translation toward a holistic model of localization that accounts for semantic, conceptual, and experiential relevance [33]. The international scope of the project also allows for comparative analysis, offering a unique opportunity to explore how socio-cultural, political, and linguistic variables shape the implementation and reception of digital mental health interventions. This is particularly important in contexts characterized by persistent traumatic stress exposure, where ongoing or repeated occupational and environmental stressors may influence patterns of help-seeking, engagement, and perceived relevance of psychological support [46]. Such findings can inform global health equity strategies and guide future adaptations in similarly complex environments [17,24]. Crucially, the study’s participatory and iterative design means that important lessons will be derived from these processes, including what works and what challenges emerge when co-producing and localizing digital interventions in diverse contexts. These lessons will be systematically captured and shared as part of the project’s dissemination strategy to support the development of future research practice in this area. This includes practical guidance for integrating trauma-informed principles, participatory methods, and cultural adaptation in digital health research and development, particularly within low-resource or conflict-affected settings.

Finally, the dissemination strategy embedded in this protocol reflects best practices in knowledge translation, ensuring that findings are communicated in ways that are accessible, impactful, and relevant across sectors. Dissemination activities will include academic publications in peer-reviewed journals, presentations at international conferences, and the development of policy briefs tailored to health system leaders and decision-makers. To ensure community reach, the research team will also produce plain-language summaries, short videos, and infographics co-designed with PPI contributors and local stakeholders. These will be disseminated through institutional websites, social media platforms, and community partner networks. In settings where digital access is limited, printed materials and community dialogues will be used to support inclusive engagement. By involving PPI contributors in shaping dissemination outputs and delivery formats, the study enhances the likelihood that its findings will be understood and used by frontline professionals, community partners, and policy audiences alike. This multi-layered dissemination approach strengthens the potential for policy influence and real-world application of research findings, particularly in contexts where digital innovation is rapidly expanding but remains unevenly accessible [15]. In sum, this protocol offers a robust and ethically grounded roadmap for the localization of digital mental health interventions. It contributes novel insights into participatory design processes, linguistic and cultural adaptation, and the practical challenges of implementing digital tools in varied global contexts. The anticipated outcomes will not only support the refinement of Sentinel but will also advance the broader field of digital mental health through evidence-based localization practices.

### Conclusions

This protocol outlines a participatory, trauma-informed, and culturally responsive approach to the co-creation and localization of a digital mental health intervention for frontline professionals in diverse global contexts. Through the integration of linguistic validation, cultural adaptation, and stakeholder engagement, the study aims to ensure that the Sentinel app is not only contextually relevant but also ethically grounded and practically useful across a range of socio-economic and linguistic settings. By developing partnerships in Saudi Arabia, South Africa, and Ukraine, the project embraces a comparative, cross-cultural lens that will yield insights into how digital mental health tools can be meaningfully adapted to meet the needs of diverse populations. The inclusion of Arabic, isiXhosa, Afrikaans, and Ukrainian translations reflects a commitment to linguistic inclusivity, while the emphasis on PPI reinforces the user-centered ethos that underpins the study. Importantly, the research will generate a rich set of insights into the practical and ethical dimensions of localization, what facilitates successful co-creation, where challenges arise, and how trauma-informed and culturally sensitive methods can be operationalized across settings. These lessons will be captured and disseminated to inform future digital mental health projects, with the goal of advancing global best practices in equitable and effective intervention design. In doing so, this research contributes to a growing evidence base on digital mental health localization and offers practical guidance for future initiatives operating in low-resource, conflict-affected, and culturally diverse settings. The anticipated outcomes (ie, enhanced engagement, acceptability, and perceived utility) will support the development of more equitable and sustainable digital mental health solutions globally. Ultimately, the study seeks to inform best practices for adapting and scaling digital interventions in ways that respect local contexts while maintaining fidelity to trauma-informed, evidence-based care.

## Abbreviations

MARS: Mobile App Rating Scale
PPI: patient and public involvement
